# Development of experimental fibrotic liver diseases animal model by Carbon Tetracholoride 

**Published:** 2017

**Authors:** Atoosa Gitiara, Samaneh Tokhanbigli, Sogol Mazhari, Kaveh Baghaei, Behzad Hatami, Seyed Mahmoud Hashemi, Ali Asadi Rad, Afshin Moradi, Meyam Nasiri, Nakisa Zarrabi Ahrabi, Mohammad Reza Zali

**Affiliations:** 1 *Basic and Molecular Epidemiology of Gastrointestinal Disorders Research Center, Research institute for Gastroenterology and Liver Diseases, Shahid Beheshti University of Medical Sciences, Tehran, Iran.*; 2 *Gastroenterology and Liver Diseases Research Center, Research Institute for Gastroenterology and Liver Diseases, Shahid Beheshti University of Medical Sciences, Tehran, Iran.*; 3 *Department of Immunology, School of Medicine, Shahid Beheshti University of Medical Sciences, Tehran, Iran*; 4 *Department of Biology, Damghan University, Damghan, Iran*; 5 *Department of Biology Central Tehran Branch, Islamic Azad University, Tehran, Iran *

**Keywords:** Liver Fibrosis, CCL4, Animal Model

## Abstract

**Aim:**

This study is presenting an effective method of inducing liver fibrosis by CCL4 as a toxin in two different breeds of rat models.

**Background:**

Liver fibrosis is a result of inflammation and liver injury caused by wound healing responses which ultimately lead to liver failure. Consequently, after liver fibrosis, the progression will be continued to liver cirrhosis and at the end stage hepatocellular carcinoma (HCC). Many studies have demonstrated that one of the most important causes of liver fibrosis is Non-alcoholic steatohepatitis (NASH). Fibrotic Liver is affected by an excessive accumulation of extracellular matrix (ECM) proteins like collagen and α-SMA.

**Methods:**

In two different experiments, male Vistar, and Sprague Dawley Rat models ranging from 200±60, corresponding to an age of approximately 10 weeks were utilized in order to induce CCL4 treated liver fibrosis.

**Results:**

After 6 weeks of CCL4 injection, different tests have been carried out to verify the liver fibrosis including serum markers such as Aspartate aminotransferase (AST) and Alanine aminotransferase (ALT), molecular tests containing, laminin and α-SMA and also pathological observation by Hematoxylin and eosin staining in both fibrosis and control group.

**Conclusion:**

The results of Pathology and Real-time PCR showed that fibrosis was induced much more effectively in Sprague Dawley rat model compared with Wistar rats.

## Introduction

 One of the substantial characteristics of the liver is self-repairing in response to injury by employing a large number of factors through different repair mechanisms. Studies have been demonstrated that the main known causes of liver failure are HCV and HBV infections, induced acute injury, alcohol abuse, and nonalcoholic steatohepatitis (NASH). NASH is a distinctive feature of type 2 diabetes and obesity which is a hard and intricate shape of non-alcoholic fatty liver disease (NAFLD), also is related to metabolic syndromes (1,2). Regarding this, NAFLD is the pre-stage in liver fibrosis gradually develop to NASH by oxidative stress leading to activation of Kupffer cells. Kupffer cells are resident macrophages in hepatic cells that are one of the participants in liver fibrosis by releasing Reactive Oxygen Species (ROS) (1,3,4). 

Another contributing factor in liver fibrosis is wound healing response to inflammation (5, 6). Liver fibrosis is defined by excessive accumulation of extracellular matrix (ECM) proteins which implicates in many related liver diseases. Over synthesis of these proteins lead to degradation of ECM structure which example of these proteins are collagen type IV, procollagen type I and III, alpha smooth muscle Actin (α-SMA), and laminin. Furthermore, up-regulation of Matrix Metalloproteinases (MMPs) and Tissue inhibitors of metalloproteinases (TIMPs) is expected, and they both play an indispensable role in an imbalance of ECM synthesis (7, 8). Precipitation of ECM proteins distorts the normal hepatic structure by forming a severe scar and further development of regenerative nodules lead to cirrhosis. Subsequently, after activation of Kupffer cells by liver injury, hepatic stellate cells (HSCs) change from quiescent to the active form and differentiate into myofibroblasts-like cells by releasing fibrogenic factors. These factors trigger the fibrotic process that includes initiation, progression, regression (9, 10) and initiation of synthesizing a large amount of ECM (2). Hepatic stellate cells (HSCs) are placed in the perisinusoidal space and are the main cells involved in liver diseases such as liver fibrosis and cirrhosis, thereby, they contribute in the formation of severe scars via response to liver damage. Hepatic Stellate Cells express the glial fibrillary acidic protein (GFAP) and desmin as neural markers and after activation they lose the capacity of storing lipid droplets and obtain a myofibroblastic phenotype which is the characteristic of upregulation of a-smooth muscle actin (11-13). 

One of the major cytokines in profibrogenic process is transforming growth factor-beta (TGF-β), which plays a pivotal role in up-regulation of α-smooth muscle actin (αSMA) and type I collagen synthesis (14). TGF-β is considered as a key player in progression of steatosis to cirrhosis and can ultimately lead to hepatocellular carcinoma. It has been hypothesized that inhibition of TGF-β could attenuate liver fibrosis. Another example of cytokines involved in progression of liver fibrosis includes Platelet-derived growth factor (PDGF) which contributes to proliferation of ECM. Studies have been showed that liver fibrosis could be a reversible process, therefore, inhibition of PDGF’s function could have a therapeutic benefit against liver fibrosis. Taken together, the necessity of investigating and clarify the exact mechanism of liver fibrosis and therapeutic approaches, it is undeniable and vitally important to have reliable animal models. In this study, we aim to establish and present an efficient protocol to induce liver fibrosis by CCL4 as a toxin reagent in rat models (15). 

## Methods

**Experimental design**

*Animals*

 7 male Wistar (Experiment one) and 3 Male Sprague Dawley (SD) (experiment two) rats weighing 200±60 grams, corresponding to an age of approximately 10 weeks were purchased from Iran university of medical sciences, acclimatized for 1 weeks in defined standard living condition, placed in 12 hours light/dark cycle, constant temperature (20 ± 2^0^C) and fed with pellet food and water. Animals were weighed before each dosing at appropriate times, and observed daily for signs of ill health. All experimental procedures were approved by the Ethical Committee of Iran University of Medical Sciences.

**Experiment one**

Rats were divided into 4 groups accepting different doses of CCL4 (Cat No: 32.022-6, Sigma, UK) dissolved in equal volume of olive oil (1:1) through intraperitoneal (IP) injection (twice a week) for 6 weeks. 3 different doses were assigned for each group (0.5, 1, 1.5 mL/kg). one group was considered as control and received the same volume of olive oil.

**Table 1:** Fibrosis score

F0	F1	F2	F3	F4
No Fibrosis	Portal areas without septa	Portal areas with few septa	Numerous septa without cirrhosis	cirrhosis
Control		●		
CCL-4 treated group			●	

**Table 2:** Primer sequences of fibrosis related genes

Gene	Primer Sequences
Procollagen I	F: 5’TCAAGATGGTGGCCGTTACT3’ R: 5’TGTTCTCAATCTGCTGGCTCA3’
Procollagen III	F: 5’GTGCAATATGTCCACAGCCT3’ R: 5’TCGCCATTTCTCCCAGG3’
Collagen IV	F: 5’GTTTCCAGGTGTAAAAGGAG3’ R: 5’CTTAAGTGTGCCAGGTTTTC3’
α-SMA	F: 5’AATGGGCCAAAAGGACAGCT3’ R: 5’TCTTTTCCATGTCGTCCCAGT3’
Laminin	F: 5’CTGCAGGAGGACAAGAAATG3’ R: 5’AGGAGCAAACGTTGTGACCA3’

**Experiment two**

 Rats were divided into two groups. The Group one (CCL4 group, n=2) received CCL4 dissolved in equal volume (1:1) of olive oil through intraperitoneal (IP) injection, subcutaneously at a dose of 1ml/kg twice weekly, for 6 weeks. The group two (n=1), received injection of olive oil vehicle only, as a negative control twice a week. At the end of the 6-week experimental period, all the rats were fasted overnight and then sacrificed. Blood was collected from the animals and the serum used for further analysis. Specimen were cut out from the liver. Half of each specimen washed with PBS to remove blood and stored at −70°C for quantitative Real Time PCR analysis. The other half was fixed in a 10% formalin solution for histopathological study.

**Determination of serum biochemical parameters**

On one hand, to verify the liver fibrosis formation, blood samples were collected into tube containing EDTA and serum separation was carried out by centrifuging at 3,000 rpm for 15 min at 4^0^C. Activity of Alanine aminotransferase (ALT), Aspartate aminotransferase (AST), and Alkaline phosphatase (ALP) were determined by Elisa assay using commercially available kits according to the manufacturer's instructions. 

**Histopathological examination**

On the other hand, parts of liver tissues were fixed in 10% paraformaldehyde and embedded in paraffin, and finally, cut into 4 µm-thick sections for histomorphological analysis. These sections were aimed for Masson’s trichrome staining to visualize collagen deposition and ECM degradation. The grades of liver fibrosis according to Ishak biopsy score have been shown in Table1.

**qRT-PCR assay for direct markers**

The Liver tissue also was used for measuring gene alteration related to fibrosis in contrast with control group such as procollagens I and III, collagen IV, α-SMA and laminin which were assessed by qRT-PCR. 

RNA of control and fibrotic livers were extracted by YTA Total RNA Purification Mini Kit (FavorGen, Taiwan) according to manufacturer’s protocol. Extracted total RNA was used in the next step for cDNA synthesis (RevertAid First Strand cDNA Synthesis Kit, cat no K1622, Thermo Scientific**)** using random hexamers in the presence of RNase inhibitor. Synthesized cDNA was applied to Real-time quantitative PCR assays. Real-Time were performed in duplicate on Rotor Gene Q Series Real-Time PCR system thermal cycler and SYBR Green Mastermix (Applied Biosystems).

Primers used to evaluate pro-inflammatory and anti-inflammatory cytokine levels were designed by primer3, NCBI. The sequences of all the primer pairs have been shown in Table 2.

**Statistical analysis**

Collected data were analyzed and the changes in mRNA expression were compared with control group with relative expression levels of targeted mRNA over the reference values. REST 2009 software version 2.0.13, and Prism 5 (One-way ANOVA analysis) were used to calculate the relative expression levels. 

## Results

24 hours after the last CCL4 injection (day 42) all rats were sacrificed. The blood sample was collected for measuring serum markers. Meantime, liver tissue discharged for evaluation of fibrosis-related genes and pathological observations.

**Experiment one**

Throughout the 6-week period of CCl4 administration, at dose levels from (0.5, 1, 1.5 mL/kg), there were no clinical signs of CCl4 toxicity. Different tests had been carried out to verify the hepatic fibrosis but did not approach any exact consequences which demonstrate the failure establishment of liver fibrosis in rats proved by histopathological observation. However, animals in the CCl4-treated groups did not gain as much weight as the control (vehicle-treated) rats.

**Experiment two**

*Pathological observation*

Throughout the 6-week period of CCl4 administration, at dose level of (1 mL/kg), the signs of CCL4 toxicity were observed and proved by histopathological observation. Fibrotic models were in the stages 3 which are defined by enlargement of stellate cells in portal tracts with numerous septa formation meaning extensive ECM degradation. In addition, the pseudolobular formation revealed the successful induction and establishment of fibrosis in CCL4-models; therefore, liver function was compromised to save retinoid.

**Figure 1:**
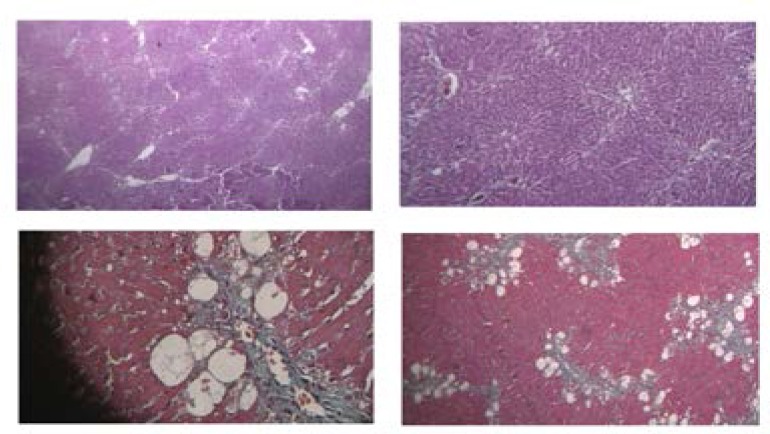
Liver histopathological observation; liver fibrosis-induced by CCL4 in rats; A: HE staining of the normal rat received olive oil, a large number of lipids with rare septa formation can be observed; B: HE staining of fibrosis models received CCL4, defined enlargement of stellate cells in portal tracts with numerous septa formation

The rat in the control group was in stage 2 and had a normal lobular architecture with central veins. However, a large number of lipids induced by olive oil were detected in control group instead of having normal lobular and structure. (Figure 1).

**Serum biochemical analysis (ALT, AST ratio)**

**Figure 2:**
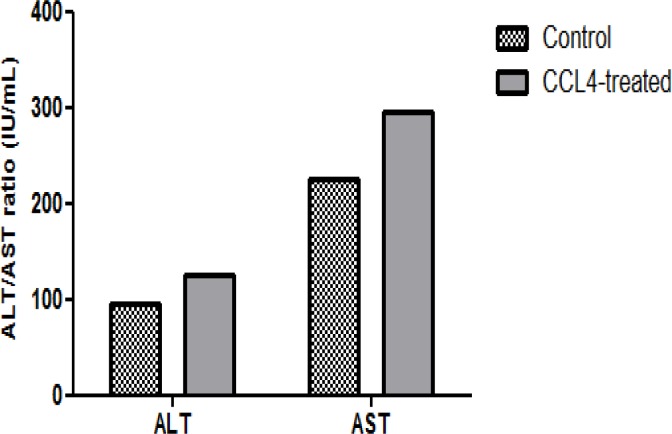
Serum biochemical parameters (ALT/ AST) analysis. Elevation of ALT and AST as a fibrotic sign in CCL4_treated group was observed

Fibrosis accumulation is a dynamic process resulting from liver injury. Aspartate aminotransferase (AST), and Alanine aminotransferase (ALT), are hepatic enzymes that are released into the bloodstream from damaged hepatocyte and upraised in the blood before the clinical signs and symptoms of liver diseases occurrence. The prognostic value of the AST/ALT ratio has been accredited in non-alcoholic liver disease, chronic viral hepatitis, primary sclerosing cholangitis, and primary biliary cirrhosis. Hence, the ratio of AST and ALT tends to elevate with stages of fibrosis. (Figure 2).

**Figure 3:**
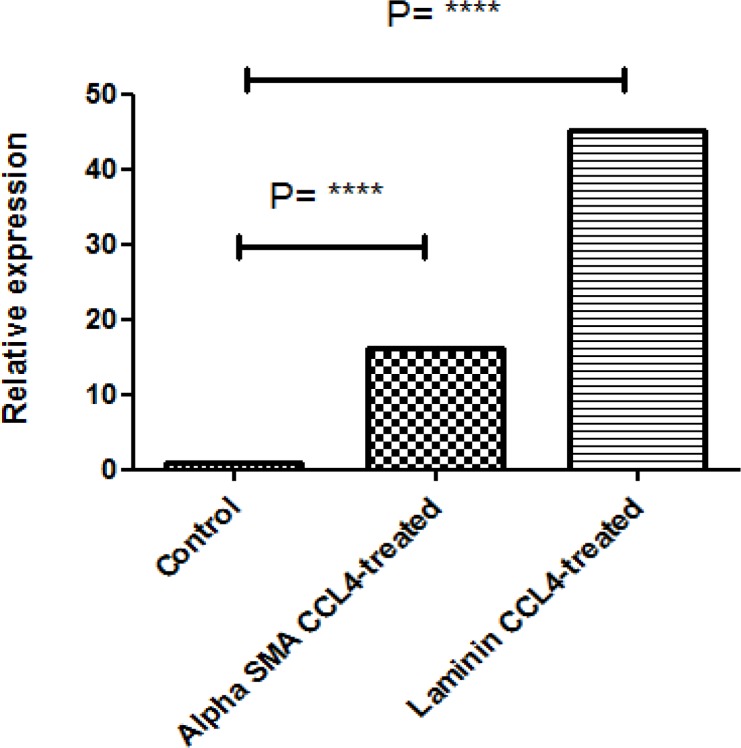
The comparative quantitative Real time-PCR for ECM degradation markers such as α-SMA and Laminin, after treatment with CCL4 compared to the control group. CCL4- treated group showed substantial elevation in both expression of Laminin and α-SMA.(P<0.0001, ANOVA

**qRT-PCR**

Liver fibrosis has many verifying test which quantitative Real Time PCR is one of them. As shown in Fig 2 the expression of Laminin and Collagen IV in CCL4 treated group were significantly increased compared to the control group. The other markers, such as α-SMA and procollagen IV relatively were elevated which demonstrated the ECM degradation in progression of liver fibrosis. (Figure 3). The comparative quantitative Real time-PCR for ECM degradation markers such as α-SMA and Laminin, after treatment with CCL4 compared to the control group. CCL4- treated group showed substantial elevation in both expression of Laminin and α-SMA. (P<0.0001, ANOVA).

## Discussion

Liver fibrosis is a dynamic process in human body representation of liver response to injury and inflammation. However, liver is known for its capability of self-renewing in serious conditions, but the process of repairing and replacing damaged cells leave a large amount of scar tissue in the liver. Fibrosis develops under constant injury and repairing which leaves severe scars in liver tissues (6). Liver fibrosis mainly is characterized by accumulation of extracellular matrix (ECM) proteins such as collagen that is the common occurrence and feature in most liver diseases (16).Therefore, over time the structure and architecture of liver and consequently the blood flow become disrupted and eventually lead to liver Cirrhosis and ultimately hepatocyte carcinoma cell (HCC) (17). Although the etiology of liver fibrosis and cellular and molecular mechanisms involved in the process are not fully elucidated, many outstanding approaches have been made in this context. Due to the importance of liver fibrosis and indispensability of this organ in human, experimental tools namely animal models in variety of human diseases are considered. Recently, substantial advances have been made in creation of reliable animal models in autoimmune diseases such as diabetes (18) and Inflammatory Bowel Disease (IBD) as gastrointestinal disease (19). However, Hepatic fibrosis models have been described in vast majority of studies by using different methods associated with toxic reagents typically CCL4 (20, 21), immunological damage (22-24), biliary fibrosis (25, 26), and alcoholic liver disease (27-29) mostly in rats and mice. Liver fibrosis and cirrhosis induced by carbon tetrachloride have advantages over the other methods that have made it the common and widely utilized method in animal models especially rats and mice. Liver fibrosis induced by CCL4 reflects a good characterization of fibrosis and the same pattern of disease observed in human caused by injury, inflammation, and all these evidence elicit the valuable models with CCL4 (30). Besides considered disadvantages CCL4 have, there is no direct human disease counterpart. In addition, the CCl4 model is the best characterized with respect to histological, biochemical, cell, and molecular changes associated with the development of fibrosis. Hence, the requirement for a CCL4 induced liver fibrosis is inevitable to investigate such a complicated disease to save many lives in the world and in this study we presented an efficient protocol to have fibrotic liver rat models in Sprague Dawley strains.
